# An Immune-Related Gene Prognostic Index for Triple-Negative Breast Cancer Integrates Multiple Aspects of Tumor-Immune Microenvironment

**DOI:** 10.3390/cancers13215342

**Published:** 2021-10-25

**Authors:** Xiaowei Wang, Wenjia Su, Dabei Tang, Jing Jing, Jing Xiong, Yuwei Deng, Huili Liu, Wenjie Ma, Zhaoliang Liu, Qingyuan Zhang

**Affiliations:** 1 Department of Medical Oncology, Harbin Medical University Cancer Hospital, Harbin Medical University, Harbin 150081, China; 201901389@hrbmu.edu.cn (X.W.); 1862@hrbmu.edu.cn (D.T.); 201801297@hrbmu.edu.cn (J.J.); 201901387@hrbmu.edu.cn (Y.D.); 2020021677@hrbmu.edu.cn (H.L.); 0178@hrbmu.edu.cn (W.M.); 2 Department of Hematology, The First Affiliated Hospital of Harbin Medical University, Harbin Medical University, Harbin 150001, China; 201901148@hrbmu.edu.cn; 3 Department of Breast Surgery, Harbin Medical University Cancer Hospital, Harbin Medical University, Harbin 150081, China; 2019021581@hrbmu.edu.cn; 4 Institute of Cancer Prevention and Treatment, Harbin Medical University Cancer Hospital, Harbin 150081, China; 5 Institute of Cancer Prevention and Treatment, Heilongjiang Province Academy of Medical Sciences, Harbin 150081, China

**Keywords:** TIME, ICI therapy, TNBC, biomarker

## Abstract

**Simple Summary:**

Triple-negative breast cancer (TNBC) is the most refractory subtype of breast cancer. Immune checkpoint inhibitor (ICI) therapy has made progress in TNBC treatment. PD-L1 expression is a useful biomarker of ICI therapy efficacy. However, tumor-immune microenvironment (TIME) factors, such as immune cell compositions and tumor-infiltrating lymphocyte (TIL) status, also influence tumor immunity. Therefore, it is necessary to seek biomarkers that are associated with multiple aspects of TIME in TNBC. In this study, we developed an immune-related gene prognostic index (IRGPI) with a substantial prognostic value for TNBC. Moreover, the results from multiple cohorts reproducibly demonstrate that IRGPI is significantly associated with immune cell compositions, the exclusion and dysfunction of TILs, as well as PD-1 and PD-L1 expression in TIME. Therefore, IRGPI is a promising biomarker closely related to patient survival and TIME of TNBC and may have a potential effect on the immunotherapy strategy of TNBC.

**Abstract:**

Tumor-immune cell compositions and immune checkpoints comprehensively affect TNBC outcomes. With the significantly improved survival rate of TNBC patients treated with ICI therapies, a biomarker integrating multiple aspects of TIME may have prognostic value for improving the efficacy of ICI therapy. Immune-related hub genes were identified with weighted gene co-expression network analysis and differential gene expression assay using The Cancer Genome Atlas TNBC data set (*n* = 115). IRGPI was constructed with Cox regression analysis. Immune cell compositions and TIL status were analyzed with CIBERSORT and TIDE. The discovery was validated with the Molecular Taxonomy of Breast Cancer International Consortium data set (*n* = 196) and a patient cohort from our hospital. Tumor expression or serum concentrations of CCL5, CCL25, or PD-L1 were determined with immunohistochemistry or ELISA. The constructed IRGPI was composed of CCL5 and CCL25 genes and was negatively associated with the patient’s survival. IRGPI also predicts the compositions of M0 and M2 macrophages, memory B cells, CD8^+^ T cells, activated memory CD4 T cells, and the exclusion and dysfunction of TILs, as well as PD-1 and PD-L1 expression of TNBC. IRGPI is a promising biomarker for predicting the prognosis and multiple immune characteristics of TNBC.

## 1. Introduction

Triple-negative breast cancer (TNBC) is one of the subtypes of breast cancer, which is named because of the negative expression of estrogen receptor (ER), progesterone receptor (PR), and human epidermal growth factor receptor 2 (HER2). TNBC accounts for about 15–20% of breast cancer cases and is associated with a high risk of mortality in the past few decades owing to its aggressiveness and the lack of effective targeted therapies [1,2,3,4].

The accumulated mutations during cancer development generated neoantigens, which make the tumor immunogenic. Immune cells can recognize neoantigens and eliminate cancer cells. However, the expression of programmed death-ligand 1 (PD-L1) in cancer cells and programmed death 1 (PD1) PD-1 in immune cells, two immune checkpoint (IC) molecules, allow cancer cells to escape the attack by immune cells, even though the immune cells have already infiltrated the tumor tissue. Immune checkpoint inhibitors (ICIs) block this immune checkpoint between cancer cells and immune cells so that the immune cells can recognize and attack cancer cells again. ICI treatments, including therapies targeting PD-1, PD-L1, and cytotoxic T lymphocyte-associated protein 4 (CTLA4), have significantly benefited the survival of many types of tumor patients [5,6,7,8,9,10].

With high levels of tumor-infiltrating lymphocytes (TILs) and PD-L1 expression, TNBC exhibits stronger immunogenicity than other subtypes of breast cancer and may be more likely to benefit from immunotherapy [10,11,12,13,14,15]. Recently, clinical trials of Impassion 130 and KEYNOTE-355 have shed a bright light on anti-PD-1/PD-L1 therapy for the treatment of TNBC patients, and the improved survival rate has become the grandest achievement in TNBC treatment in a decade [16,17].

Some breast cancer patients are not sensitive to PD-1/PD-L1 treatment [18]. In other cancer types, several criteria are used to evaluate whether a patient would benefit from anti-PD-1/PD-L1 therapy, including PD-L1 expression, tumor mutation burden (TMB), and microsatellite instability (MSI), which detects mismatch repair defects [19,20]. However, mismatch repair defects and high TMB occur at a low frequency in breast cancer [19,21], leaving PD-L1 expression as the only biomarker in TNBC currently. However, PD-L1 as the ICI treatment biomarker also has some limitations. For example, different PD-L1 detection methods will affect the consistency of detection results [22], and some PD-L1-negative patients also showed a response to ICI treatment [23]. Tumor-immune microenvironment (TIME) has long been shown to have an impact on the survival of TNBC patients. The composition of immune cells in the TIME of TNBC, as well as the status of TILs and cancer cells, may all have an impact on the outcome of ICI therapy. Therefore, it is of great interest to explore novel predictive indicators related to TIME to improve the efficacy of immunotherapy in TNBC patients.

Chemokines are a family of small, secreted proteins that bind to their G protein-coupled heptahelical receptors on the cell surface. The primary role of chemokines is to stimulate inflammatory cell migration, thus involving immune and inflammatory responses. According to the *n*-terminus of chemokines, they are divided into four subfamilies: CC, CXC, CX3C, and XC [24,25]. As the largest subfamily, CC chemokines have been reported as crucial players of TIME and immune response, as well as tumor growth and progression [26,27,28].

In this study, we constructed a prognostic signature composed of the CC chemokines to predict TNBC prognosis and immune characteristics. We focused on all immune-related genes in the transcriptome data of TNBC and screened immune-related hub genes related to patient prognosis by weighted gene co-expression network analysis (WGCNA). An immune-related gene prognostic index (IRGPI) was constructed, and its prognostic value was confirmed with multiple cohorts. Its relationships with the profiles of tumor-immune cells, the status of TILs and PD1/PD-L1 immune checkpoints were further characterized. The results show that IRGPI was a promising marker for predicting the prognosis and TIME status in TNBC.

## 2. Materials and Methods

### 2.1. Patients and Data Sets

RNA-seq data (Level 3) of breast cancer patients were obtained from the TCGA database. Triple-negative breast cancer (TNBC) data were extracted based on estrogen receptor (ER), progesterone receptor (PR), and proto-oncogene HER-2 status, and only patients with overall survival (OS) > 30 days were selected. The RNA-seq data included 115 cancer samples and 13 adjacent normal tissue samples. Clinical information (including survival time and status, age, TNM, and stage) of patients with TNBC was obtained from UCSC Xena (http://xena.ucsc.edu/, accessed on 11 January 2021). We also downloaded the immune-related genes from the ImmPort database (https://www.immport.org/home, accessed on 11 January 2021) and the InnateDB (https://www.innatedbdb.com, accessed on 11 January 2021) database.

The gene expression and clinical data of the Molecular Taxonomy of Breast Cancer International Consortium (METABRIC) data set were downloaded from the cbioportal website, and 196 TNBC patients with OS > 30 days were selected for the model validation.

### 2.2. WGCNA

Weighted gene co-expression network analysis (WGCNA) is a powerful tool for finding clusters (modules) of highly interconnected genes [29]. WGCNA was performed to identify co-expression modules of immune-related genes obtained from ImmPort and InnateDB. First, the co-expression similarity matrix was constructed by calculating the Pearson correlation coefficient between two genes. Next, using the scale-free topology criterion R^2^ = 0.9, a soft threshold of β = 4 was picked and used to calculate the signed adjacency matrix from the similarity matrix. With the dendrogram cut height for module merging set to 0.25, we identified 7 modules. Each color module represented a collection of genes that are highly correlated to each other among the patients, except the gray module. Genes not to cluster with any modules were assigned into the grey module. A topological overlap matrix (TOM) was used to visualize the gene–gene connectivity.

The genes in each module (except the gray module) were subject to Gene Ontology (GO) and Kyoto Encyclopedia of Genes and Genomes (KEGG) analyses with the clusterProfiler package of R to identify significantly enriched pathways. CC chemokines play an important role in inflammatory response and immunity [27]. The modules containing CC chemokine family members were the modules we were interested in (blue and brown modules) and were selected and used for the subsequent analyses. In the blue and brown modules, the edges between two genes with a weight >0.2 were used to construct the networks of genes in the modules.

### 2.3. Identification of Differentially Expressed Genes

Based on the RNA-seq data of TCGA TNBC samples (115 tumors vs. 13 normal samples), differentially expressed genes (DEGs) (adjusted *p*-value < 0.05, |log2(Fold Change)| > 1) were identified using the edgeR package of R. Next, the DEGs in the blue and brown modules were identified as immune-related DEGs for subsequent analysis.

### 2.4. Construction and Validation of the Immune-Related Gene Prognostic Index (IRGPI)

First, univariate Cox regression analysis was used to determine associations between immune-related DEGs and overall survival in TNBC patients, and genes with *p* values < 0.05 were defined as survival-related genes. Then, the survival-related genes were used to construct an IRGPI model using the multivariate Cox regression (R “survival” package) and stepwise regression analysis (R “stats” package). The coefficients of the final optimal regression model were calculated by the stepwise regression analysis. We then calculated prognostic indexes for all the cancer samples by the formula IRGPI = expression level of gene1* coef1 + expression level of gene2* coef2 +…+ expression level of geneN* coefN. TCGA patients were divided into an IRGPI-high group and an IRGPI-low group according to the median IRGPI score. KM survival curves were used to evaluate the IRGPI model with the log-rank test in the TCGA cohort, which was further validated with the METABRIC cohort. The prognostic model of survival was further evaluated by calculating the AUC values (the areas under the ROC curves) at 1, 3, 5, and 7 years. We also performed the multivariate Cox regression analysis using important clinicopathological features (age, TNM, and stage) and IRGPI scores in the TNBC patients.

### 2.5. Identification of Molecular Characteristics between Different IRGPI Groups

Differential expression analysis was first performed for the groups with high (*n* = 57) and low (*n* = 58) IRGPI scores. The DEGs in the IRGPI-high and low groups were subjected to the enrichment analysis and the gene set enrichment analysis (GSEA) to determine the signaling pathways involved with the clusterProfiler package of R (*p*-value < 0.05).

### 2.6. Determine the Levels of Immune Cell Infiltration between Different IRGPI Groups

CIBERSORT is a method using gene expression profiles to characterize cell compositions of complex tissues [30]. We used LM22, a leukocyte gene signature matrix containing 547 genes that distinguish 22 human hematopoietic cell phenotypes to determine the levels of immune cell infiltration in TCGA TNBC samples. We obtained the CIBERSORT R script from CIBERSORT website (https://cibersort.stanford.edu/, accessed on 11 January 2021) and analyzed the mRNA expression matrix in combination with LM22 in TNBC samples. The Wilcoxon rank sum test was used to calculate the levels of immune cell infiltration among different IRGPI groups (*p* values < 0.05), and Spearman correlation was used to calculate the correlation between immune cells (*p* values < 0.05).

### 2.7. GO and KEGG Analyses of Immune-Related DEGs

Immune-related DEGs were obtained and analyzed using GO and KEGG analyses with the clusterProfiler package of R to analyze the biological processes (BP), cellular component (CC), and molecular function (MF) of the DEGs involved and to identify significantly enriched pathways (adjusted *p* values < 0.05).

### 2.8. TIDE Analyses in Different IRGPI Groups

TIDE is a computational method to evaluate T-cell dysfunction and exclusion in tumor microenvironments [31]. We used TIDE to assess the individual response to immunotherapy, and the Wilcoxon rank sum test was used to calculate the difference in TIDE scores between different IRGPI groups (*p* values < 0.05). In addition, Spearman correlation was also adopted to investigate the correlation between prognostic markers (IRGPI, CCL5, and CCL25) and PD-L1/PD1 expression.

### 2.9. Specimen and Clinical Data

Formalinfixed, paraffin-embedding (FFPE) specimens were collected from the Harbin Medical University Cancer Hospital. Tissues were collected from 89 TNBC patients who underwent radical mastectomy of breast cancer between 2012 and 2015 and 40 patients between 2020 and 2021 in Harbin Medical University Cancer Hospital. Serum samples were collected from the 40 surgical patients diagnosed between 2020 and 2021 mentioned above. The patients did not have hepatitis and other infectious diseases or immune diseases. The study was approved by the Ethics Committee of Harbin Medical University Cancer Hospital. Follow-up time ranged from 1 to 107 months, with a median of 72 months.

### 2.10. Immunohistochemistry

Tissue sections were dewaxed in xylene and hydrated gradually through graded alcohol. EDTA buffer was used for antigen retrieval. The endogenous peroxidase activity was blocked, and then the sections were incubated with the primary anti-CCL25 (1:200; ab200343, Abcam, Cambridge, UK), anti-CCL5 (1:100; ab9679, Abcam, Cambridge, UK), or anti-PD-L1 (1:100; SK006, DAKO) overnight at 4 °C. Then the secondary antibody and a DAB kit (K5007, DAKO; Dako REAL™ EnVision™) were applied to the sections. A rabbit-specific HRP/DAB (ABC) IHC Detection Kit (ab64261, Abcam, Cambridge, UK) was used for CCL25. Normal tonsil tissue was used as a positive control for CCL5 and PD-L1 IHC, and normal thymus tissue was used as a positive control for CCL25 IHC (Figure S4).

The tissue sections were evaluated by two pathologists who were unaware of the patient’s clinical information. Positive PD-L1 expression was defined as the Combined Positive Score (CPS) ≥ 10 [32,33]. For CCL5 and CCL25 IHC, the scores were assigned from 0 to 3 based on the staining intensity level (no staining, light brown, brown, and tan). The staining extent was graded from 0 to 4 for the percentage of positive cells (0–5%, 5–25%, 26–50%, 51–75%, and 76–100%). The product of staining intensity and extent scores was used as the IHC score, with a range of 0 to 12. Scores 0–4 were assigned as negative, and scores 5–12 were assigned as positive.

### 2.11. ELISA

Serums CCL5 and CCL25 were measured by ELISA (Cloud-Clone Corp, Wuhan, China) according to the manufacturer’s instructions. Optical densities at 450 nm of replicate specimens were determined in a plate reader. Untreated wells were used as the blank control.

### 2.12. Statistical Analysis

All statistical analysis was performed with R language. χ^2^ test was used to compare the relationships between the IRGPI and clinicopathological factors of TNBC, and the Cox regression analysis was used to determine hazard ratios (HRs) and 95% confidential intervals (CIs) for univariable and multivariable analyses. Progression-free survival (PFS) was defined as the time between the initiation of surgical treatment and the date of the first evidence of tumor progression. We used Kaplan–Meier plots and log-rank tests to calculate the differences in PFS among different subgroups. The Pearson correlation analysis was used to calculate the correlation of two variables. *p* < 0.05 was defined as a statistical significance.

## 3. Results

### 3.1. Identify Gene Co-Expression Networks of Immune-Related Genes in TNBC Samples

**Figure 1:**
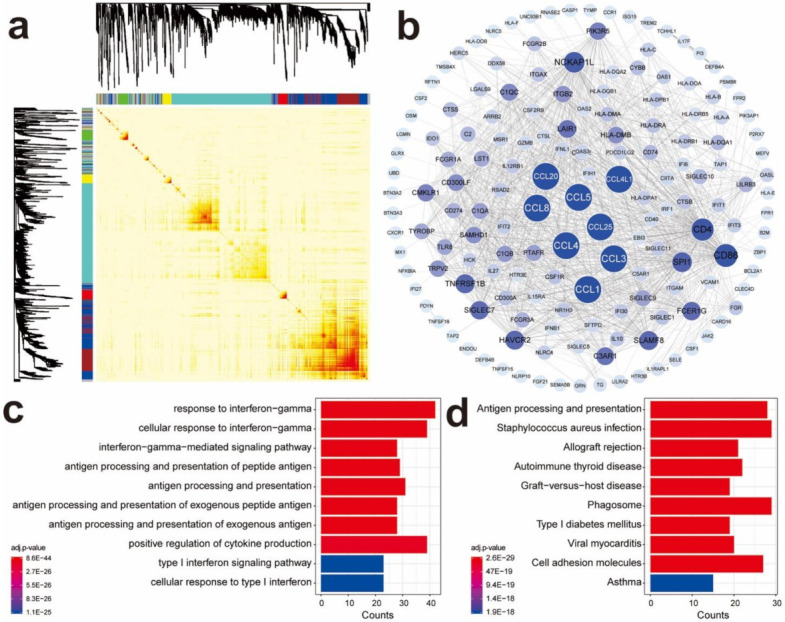
Identifying co-expressed immune genes related to CC chemokine genes by WGCNA. (**a**) Heat map of the Topological Overlap Matrix in WGCNA analysis; (**b**) co-expression network of genes in the blue module; (**c**) GO enrichment of genes in the blue module of co-expression network; (**d**) KEGG enrichment of genes in the blue module of co-expression network.

To obtain the immune-related hub genes, WGCNA analysis was carried out on the immune genes obtained from the ImmPort and InnateDB databases. The WGCNA analysis identifies gene clusters that are highly correlated to each other among patients. The results are shown with modules of different colors. Seven modules were then identified with a soft threshold of 4. A total of 1957 genes were allocated to 7 modules (432 in blue, 282 in brown, 77 in green, 72 in red, 842 in turquoise, 102 in yellow, and 150 in grey) (Figure 1a and Figure S1b). With the GO and KEGG analyses, we found that the CC chemokine family genes were significantly enriched in the blue and brown modules (19/26, 14 in blue, 5 in brown, 3 in turquoise, 1 in green, 1 in red, and 2 in grey). Therefore, the genes in the blue and brown modules (including 432 genes in the blue module and 282 genes in the brown module) were selected for further analysis (Table S1). The analysis of co-expression networks of genes in the blue and brown modules identified 162 genes and 1169 edges in the blue module (Figure 1b) and 196 genes and 7151 edges in the brown module (Figure S1c) with a threshold weight >0.2. The 358 genes were subject to GO and KEGG analyses, and the top ten significantly enriched GO terms and KEGG pathways are separately shown (Figure 1c,d and Figure S1d,e).

### 3.2. Identify Differentially Expressed Immune-Related Hub Genes in TNBC Samples

**Figure 2:**
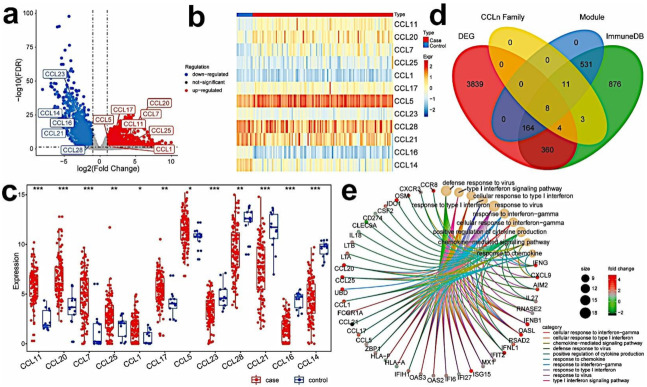
Identifying immune-related hub genes that are differentially expressed in tumor and normal samples. (**a**) Volcano plot of DEGs in TNBC and normal samples; (**b**,**c**) heat map and box plot of differential CC chemokines genes between TNBC (red) and normal samples (blue) (ns: not significant, * *p* < 0.05; ** *p*< 0.01; *** *p* < 0.001); (**d**) Venn diagram of immune-related genes, DEGs in TNBC and normal samples, genes in the blue and brown modules, and genes of CC chemokines. (**e**) GO enrichment of immune-related hub DEGs (Top10).

By comparing the expression data of 115 tumors vs. 13 normal samples, a total of 4375 DEGs were obtained, of which 2377 genes were upregulated, and 1998 genes were downregulated in the tumor samples (Figure 2a, Table S2). Interestingly, 12 of 28 CC subfamilies of chemokines were differentially expressed. Among them, CCL11, CCL20, CCL7, CCL25, CCL1, CCL17, and CCL5 were significantly upregulated, while CCL23, CCL28, CCL21, CCL16, and CCL14 were significantly downregulated (Figure 2b,c). The DEGs in normal and tumor samples were further intersected with the 358 genes obtained from the blue and brown modules; 67 genes were attained as differentially expressed immune-related hub genes (Table S3). GO and KEGG function enrichment analysis was performed on these hub genes, and the results show that these key genes were significantly associated with 389 GO terms (353BP + 12CC + 24MF) and 41 KEGG pathways (Table S4). The relationship between the 67 hub genes and top 10 GO categories are illustrated in Figure 2e. In addition, six of the differentially expressed CC subfamily chemokines, including CCL17, CCL21, CCL5, CCL1, CCL25, and CCL20, were in the hub gene list.

### 3.3. Establish the Immune-Related Gene Prognostic Index (IRGPI) in TNBC

**Figure 3:**
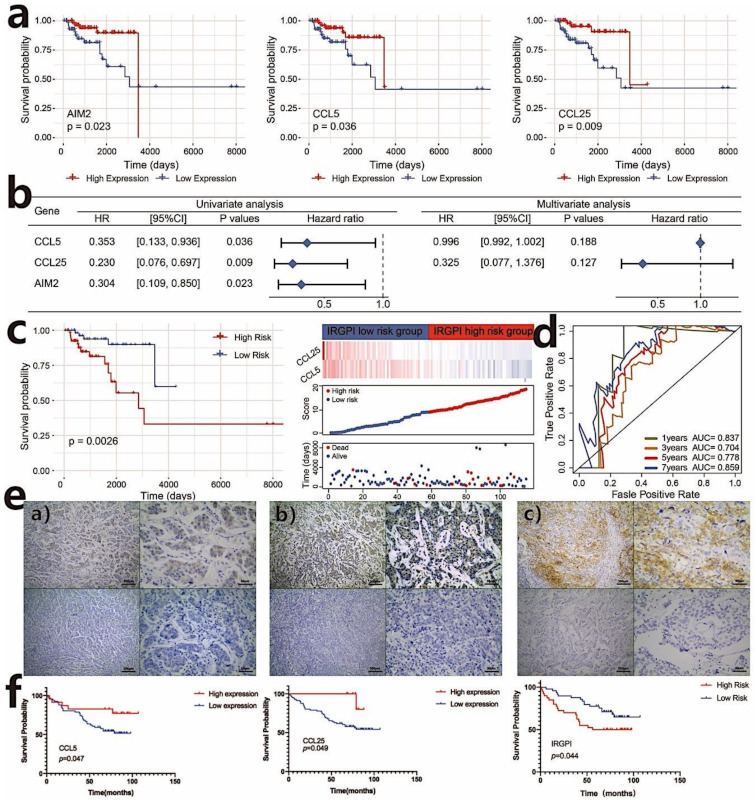
Construct of the immune-related gene prognostic index (IRGPI) in TNBC. (**a**) Kaplan–Meier survival analysis of AIM2, CCL5, and CCL25 genes that are significant in the univariate Cox analysis (*p* < 0.05); (**b**) univariate and multivariate Cox regression analysis of survival-related genes; (**c**) Kaplan–Meier survival analysis of IRGPI scores using TCGA data and the relationship between the survival status and IRGPI score distribution in the TCGA TNBC cohort; (**d**) ROC curves of the prognostic models in TNBC (the TCGA cohort) at 1, 3, 5, and 7 years; (**e**) expression of CCL25, CCL5, PD-L1 in TNBC. (**a**) Representative samples with CCL25 positive (above) and negative (below) expression; (**b**) representative samples with CCL5 positive (above) and negative (below) expression; (**c**) representative samples with PD-L1 positive (above) and negative (below) expression. Scale bars: 200 μm for the left pictures in (**a**–**c**), and 50 μm for the right pictures in (**a**–**c**); (**f**) Kaplan–Meier survival analysis of CCL5 and CCL25 expression and IRGPI groups using IHC scores of the patients from our hospital (*p* < 0.05).

To investigate the survival outcomes of the 67 immune-related hub genes, univariate and multivariate Cox regression analyses for OS were performed. Three genes, including AIM2, CCL5, and CCL25, were significantly associated with the OS of TNBC patients in univariate Cox regression analyses (Figure 3b). Figure 3a shows the Kaplan–Meier (KM) plots of AIM2, CCL5, and CCL25. The three candidate predictive variables were subject to the stepwise regression analysis, and the coefficients of the final optimal regression model were calculated. The IRGPI of each sample was then calculated by the final model equation: IRGPI = expression level of CCL25 × (−1.1233) + expression level of CCL5 × (−0.0033). The KM survival curves showed that TNBC patients with a low IRGPI had better OS than the patients with a high IRGPI (*p* = 0.0026, log-rank test) (Figure 3c). The predictive performance of this prognostic model was evaluated by ROC curves, and the AUC for 5-year survival was 0.778, showing a good predictive ability. In addition, the ROC curves for 1-year, 3-year, 5-year and 7-year survival are shown in Figure 3d.

Next, we performed the multivariate Cox regression analysis with available TNBC clinical information (AGE, TNM, and STAGE) and IRGPI scores to determine whether IRGPI was an independent prognostic predictor of overall survival. Univariate Cox regression analysis showed that the IRGPI group was significantly associated with overall survival (HR = 1.139, 95%CI = 1.043–1.243, *p* values= 0.004). Multivariate Cox regression analysis confirmed that IRGPI was an independent prognostic factor after adjusting for other clinicopathologic factors (HR = 1.138, 95%CI = 1.040−1.244, *p* values = 0.005) (Figure S2a).

### 3.4. Validation of the Prognostic Value of IRGPI

We further validated the prognostic value of CCL5, CCL25, and the prognostic model with other cohorts. We used the TNBC cohort of the Kaplan–Meier plotter database (http://kmplot.com/, accessed on 21 February 2021) to evaluate the effect of CCL5 and CCL25 expression on survival. The results show that CCL5 expression was positively associated with the overall survival (OS) of TNBC patients (log-rank test, *p* values < 0.05). Similarly, the OS of the high-CCL25-expression group was superior to the low-CCL25-expression group (log-rank test, *p* = 0.064) (Figure S2b). Because the prognostic value of IRGPI could not be validated with the Kaplan–Meier plotter database, we further analyzed IRGPI scores in the TNBC cohort of the METABRIC data set (*n* = 196). Consistent with the result of the TCGA data set, the IRGPI-low subgroup had a significantly better prognosis than those in the IRGPI-high subgroup (*p =* 0.049, log-rank test) (Figure S3a).

We then further validated the association of IRGPI with PFS in TNBC patients from our hospital. The 89 TNBC patients with a median PFS of 72 months (ranging from 1 month to 107 months) underwent radical mastectomy after being diagnosed with breast cancer between 2012 and 2015. To confirm the correlation of IRGPI with the characteristics of TNBC patients, we used the semi-quantitative IHC scores of CCL5 and CCL25 to calculate IRGPI in the TNBC patients from our hospital. As shown in Figure 3e, CCL5 was expressed in cancer cells and lymphocytes, while CCL25 was mainly expressed in cancer cells. Consistent with the TCGA results, a high expression of CCL5 (*p* = 0.047) and CCL25 (*p* = 0.049) was associated with a better PFS. Furthermore, the KM estimator showed that PFS in the IRGPI-low group was significantly longer than that in the IRGPI-high group (HR = 0.508; 95% CI, 0.263–0.981; *p* = 0.044). Because age was significantly associated with PFS in the univariate analysis, it was added to IRGPI for multivariate COX analysis, and the results also show that IRGPI was an independent biomarker for evaluating the outcomes of patients (HR = 0.470; 95%CI, 0.242–0.912; *p* = 0.026) (Figure 3f, Table 1 and Table 2).

### 3.5. Identifying Differentially Expressed Genes Associated with IRGPI Status

**Figure 4:**
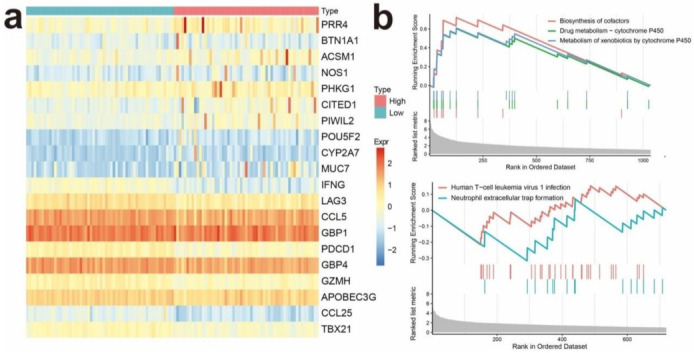
Identifying differentially expressed genes (DEGs) in different IRGPI subgroups. (**a**) Heat map of DEGs between high and low IRGPI risk groups; (**b**) significantly enriched pathways of upregulated DEGs in the high (above) and low (below) IRGPI risk groups revealed by GSEA.

Differentially expressed genes in the IRGPI groups showed that 1096 genes were upregulated in the IRGPI-high group while 750 genes were downregulated (Figure 4a, Table S5). The top 10 significantly upregulated and downregulated genes are shown in Figure 4a (Figure 4a, Table S5), and both CCL5 and CCL25 are among the top 10 significantly downregulated genes in the IRGPI-high group. GSEA was performed to determine the gene sets enriched in different IRGPI subgroups. The gene sets upregulated in the IRGPI-high samples were enriched with 10 pathways, including the biosynthesis of cofactors, drug metabolism-cytochrome P450, and metabolism of xenobiotics by cytochrome P450, while the gene sets upregulated in the IRGPI-low samples were enriched with neutrophil extracellular trap formation and human T-cell leukemia virus 1 infection pathways (Figure 4b).

### 3.6. IRGPI Is Associated with Immune Cell Compositions in TNBC Samples

**Figure 5:**
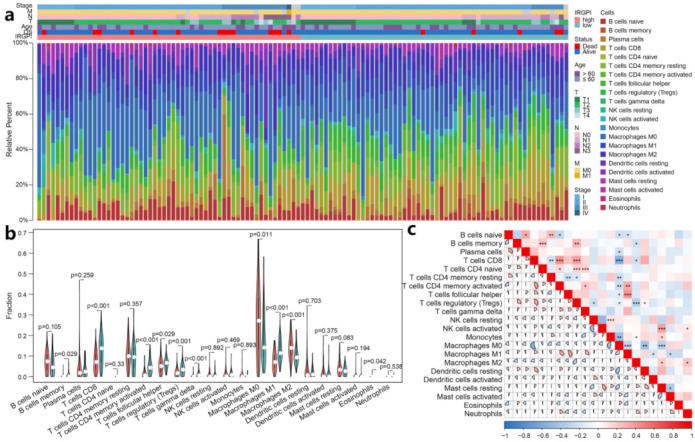
Immune cell compositions in TIME of different IRGPI groups. (**a**) The proportions of 22 tumor infiltrated immune cells of individual patient calculated by CIBERSORT; (**b**) the difference of immune cell infiltration in different IRGPI groups (pink: high IRGPI risk group; and blue: low IRGPI risk group); (**c**) correlations among immune cells in TNBC (ns: not significant, * *p* < 0.05; ** *p* < 0.01; *** *p* < 0.001).

To analyze the composition of immune cells in different IRGPI subgroups, CIBERSORT was used to systematically evaluate the infiltration level of immune cells in each sample, and the Wilcoxon test was used to compare the distribution of immune cells between different IRGPI groups (Figure 5a and Table S6). We found that M0 macrophages, M2 macrophages, and eosinophils were more abundant in the IRGPI-high subgroup, while memory B cells, CD8^+^ T cells, activated memory CD4 T cells, follicular helper T cells, regulatory T cells (Tregs), gamma delta T cells and M1 macrophages were more abundant in the IRGPI-low subgroup (Figure 5b). In addition, the association between immune cells in TNBC indicated the interactions inside immune microenvironments (Figure 5c).

Next, we verified this result with the METABRIC data set. Consistent with the TCGA results, M0 macrophages and M2 macrophages were also more abundant in the IRGPI-high subgroup, and there were more memory B cells, CD8^+^ T cells, activated memory CD4 T cells in the IRGPI-low subgroup (Figure S3b). Similarly, M1 macrophages also tended to be enriched in the IRGPI-low subgroup (*p* = 0.087). Besides, the compositions of plasma cells, naive CD4 T cells, activated NK cells and activated mast cells were also significantly higher in the IRGPI-low subgroup than the high subgroup, but activated master cells and neutrophils were markedly low in the IRGPI-low subgroup. Even though the differences exist between the two data sets, the results of major TIME players are consistent, including memory B cells, CD8^+^ T cells, activated memory CD4 T cells, M0, M1, and M2 macrophages.

### 3.7. IRGPI Is Associated with T-Cell Exclusion and Dysfunction in TNBC Samples

**Figure 6:**
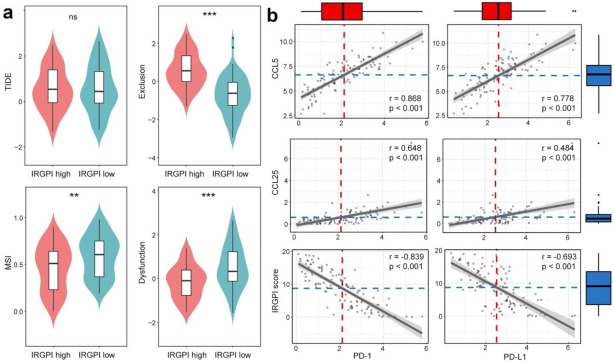
IRGPI is associated with T-cell exclusion and dysfunction, and PD1 and PD-L1 expression in TNBC of TCGA data set. (**a**) Scores of TIDE, MSI, and T-cell exclusion and dysfunction in different IRGPI risk groups (ns: not significant, ** *p* < 0.01; *** *p* < 0.001); (**b**) Correlation between CCL5/CCL25/ IRGPI scores and PD-L1/PD1.

We then used TIDE to assess the potential treatment efficacy of immunotherapy in different IRGPI subgroups (Table S7). A higher TIDE prediction score represented a higher potential for immune evasion, which suggested that the patients were less likely to benefit from ICI therapy. The IRGPI-low subgroup was not associated with TIDE scores (*p* = 0.99) in the TCGA data set (Figure 6a). However, we found that the IRGPI-low subgroup had a higher T-cell dysfunction score, but a lower T-cell exclusion score (Figure 6a). The microsatellite instability (MSI) score of IRGPI-low subgroup is higher than the IRGPI-high subgroup in the TCGA data set.

Similar results were also observed in the METABRIC data set. The IRGPI-low subgroup had a higher T-cell-dysfunction score, but a lower T-cell-exclusion score. Unlike the TCGA data set analyses, the IRGPI-low subgroup was also significantly associated with a low TIDE scores (*p* = 0.025) and a lower MSI score in the METABRIC analyses (Figure S3c). These results demonstrate that IRGPI performs well in predicting T-cells dysfunction and exclusion in the TNBC samples.

### 3.8. IRGRI Is Correlated with PD-1 and PD-L1 Expression in TNBC Samples

Because PD-L1 expression was the most frequently used criterion to determine whether the patients should undergo ICI therapy for multiple cancers, we explored the correlation of PD-L1/PD1 expression with IRGPI scores, as well as with the expression of CCL5 or CCL25. The results show that the expression of CCL5 and CCL25 were positively correlated with the expression of PD-L1 and PD1, while the IRGPI score was negatively correlated with the expression of PD-L1 and PD1 in both TCGA and METABRIC data sets (Figure 6b and Figure S3d).

To confirm the correlation of IRGPI with PD-L1 expression in TNBC, we used the IHC results to verify. In the 129 TNBC patients from our hospital, both CCL5 (r = 0.179, *p* = 0.043) and CCL25 (r = 0.203, *p* = 0.021) expression were correlated with PD-L1 expression (Table 3). Next, we analyzed the relationship between IRGPI and PD-L1 expression. The results show that IRGPI was negatively correlated with PD-L1 expression (r = −0.204; *p* = 0.020), indicating that PD-L1 expression was higher in IRGPI-low group (Table 4).

Non-invasive tests are always favorable in clinics. Therefore, we also examined the correlation between tumor tissue PD-L1 expression and serum IRGPI, as well as the serum levels of CCL5 or CCL25. In the 40 TNBC patients who had both serum and tumor tissue available, we found a negative correlation between serum IRGPI and tissue PD-L1 expression, as well as between serum CCL25 levels and tissue PD-L1 expression, although the correlation was not statistically significant (r = −0.302; *p* = 0.058) (Table 4 and Table 5).

## 4. Discussion

TIME, which are affected by numerous genes, are critical for tumor growth and ICI therapy effects. Chemokines are pivotal molecules that regulate the migration of immune cells, and may thus shape the TIME in TNBC. To reduce the complexity of the gene co-expression network, we employed WGCNA to cluster immune-related genes and identify immune-related hub biomarkers in chemokine enriched modules. The 67 immune-related hub genes were further subject to survival analysis, and an immune-related gene prognostic index (IRGPI) was constructed, composed of two CCL genes, CCL5 and CCL25. IRGPI was further demonstrated to perform superbly as an independent prognostic factor for TNBC in multiple cohorts, including TCGA, METABRIC, and a cohort of patients from our hospital. IRGPI predicts better survival outcomes for IRGPI-low patients and worse outcomes for IRGPI-high patients. The semi-quantitative IHC scores obtained from the patients of our hospital also confirmed similar results. The consistency among different cohorts indicates the great prognostic value of the IRGPI and suggests the component of IRGPI may be critical for the modulation of TIME in TNBC.

The role of CCL5 and CCL25 is not well understood in TNBC. CCL5 is mainly secreted by T lymphocytes, macrophages, platelets, synovial fibroblasts, tubular epithelium, and tumor cells [34,35,36,37]. The expression of CCL5 and its receptor CCR5 has been found to be elevated in many tumors, including triple-negative breast cancer [38,39,40,41,42,43,44,45,46,47,48,49,50,51,52,53]. In our study, we have found CCL5 is mainly expressed in cancer cells and tumor-associated lymphocytes. CCL5 and its receptor have been reported to be associated with the progression and drug resistance of many tumors, including breast cancer [54,55,56,57]. However, CCL5 is a double-edged sword in cancer. CCL5 also enhances anti-tumor immunity and promotes immunotherapy by recruiting anti-tumor-immune cells to the tumor microenvironment [26,27,58,59,60]. In our study, we found that a high expression of CCL5 was associated with the better survival of TNBC patients in three different cohorts. In bc-GenExMiner (V4.1 DNA chip database), the univariate Cox regression analysis also showed that CCL5 (*p* = 0.0335, HR = 0.94) in metastatic relapse breast cancer had a positive effect on prognosis [61]. All these results suggest a positive effect of CCL5 on TNBC prognosis.

CCL25, also known as thymus-expressed chemokine (TECK), is the ligand for CCR9 [24]. CCL25 is produced by tumor-associated cells or cancer cells, such as breast cancer cells and pancreatic cancer cells [62,63]. Previously, Chen et al. [64] showed that CCL25 was not expressed in TNBC tumors by IHC. However, our study showed that although the expression of CCL25 was low, CCL25 was expressed in TNBC tumors, primarily in cancer cells. In lung cancer, the CCL25/CCR9 axis promotes cancer progression [64,65,66], but intratumoral delivery of CCL25 attracts CCR9^+^ CD8^+^ T cells to infiltrate the tumor and enhances CD47-targeted immunotherapy in a murine TNBC model [64]. Thomas et al. [61] consistently found that the high CCL25 (*p* = 2.7 × 10^−6^, HR = 0.77) expression in breast cancer was associated with an increase in RFS (relapse-free survival). Similarly, the neutralization of CCL25 also promoted tumor growth in a CCL25-expressing mouse melanoma model [67]. Our study further confirmed the favorable effect of CCL25 on TNBC prognosis.

AIM2 expression showed a significant association with TNBC survival in the univariate analysis but was not included in the final IRGPI score. AIM2 is a component of inflammasome, which plays a crucial role in the function of T regulatory cells [68]. Its role in cancer microenvironements is an intriguing topic that requires more investigation. That it was not included in the IRGPI score could possibly be attributed to the following reasons. First, it may be related to the process of stepwise regression. During the analysis, the candidate variables were added or removed into the cox regression analysis in a stepwise manner. To obtain the optimal final model, the analysis evaluated the predictive power of the model in every step until there was no justifiable reason to add or remove any more. Mathematically, if adding AIM2 did not increase the statistical power for the final model, it would not be included in the final model. Second, biologically, AIM2 is a gene highly expressed in Tregs. Its expression and function may be affected by factors regulating Tregs and the infiltration levels of Tregs in the tumor. Thus, its predictive power might be overridden by other factors in the multivariate analysis.

Owing to the crucial role of CCL5 and CCL25 in TIME, we analyzed the immune cell profiles in TNBC to explore the link between IRGPI and tumor-immune cell compositions. The composition of immune cells differed between two IRGPI subgroups. Cytotoxic CD8 T cells, CD4 T cells, and M1 macrophages were more enriched in the IRGPI-low subgroup, and M0 and M2 macrophages were more abundant in the IRGPI-high subgroup. A large number of studies have shown that dense infiltration of T cells, especially cytotoxic CD8 T cells, indicates a favorable prognosis [69,70,71]. This is in line with previous clinical observations in TNBCs [72]. In most tumors, M2 macrophages have been proven to favor the development of tumor. In breast cancer, M2 macrophages inside tumors promote immunosuppression and predict a poor outcome. Conversely, M1 macrophages are known to exert anti-tumor activity by promoting immune responses, thus predicting a favorable prognosis in many cancers [70,73,74,75,76]. These results suggested that the IRGPI-low subgroup had a favorable immune microenvironment than the IRGPI-high subgroup.

The function of infiltrating cytotoxic T lymphocytes (CTLs) is not only related to their levels but also to their appropriate priming. A new algorithm, TIDE, was recently developed to model tumor-immune evasion by evaluating the exclusion level of T cells, as well as the priming level of infiltrating CTLs [31]. TIDE exhibits a better performance in evaluating the efficacy of first-line ICI therapy in melanoma patients compared with the widely used biomarkers for ICI therapy, such as tumor mutation burden and PD-L1 expression. We show here that the CTL exclusion level was consistently low in the IRGPI-low groups in both TCGA and METABRIC data sets. This observation agrees with the CIBERSORT analysis, in which the CD8^+^ T cells infiltrated the IRGPI-low groups at a significantly higher rate. However, the high CTL dysfunction scores in the IRGPI-low TNBC suggests a compromised cytotoxic response to cancer cells due to immune checkpoints modulated by PD-L1 and PD-1 molecules and thus a potential benefit from ICI therapy for the IRGPI-low patients. We noticed that TIDE and MSI scores were not always associated with IRGPI. This may be attributed to the inherent data structure differences in gene expression of the two data sets: TCGA gene expression data are RNA-seq results, which are more accurate and have broader detection ranges, whereas the METABRIC gene expression data are from microarrays, which may exhibit false hybridization and hybridization saturation problems. Nevertheless, the inconsistent results suggest more care should be taken when applying TIDE scores in TNBC. And since the frequency of MSI in breast cancer is relatively low, it also should not be used in evaluating ICI therapy efficacy in TNBC.

Next, we examined the relationship between PD-1/PD-L1 expression and IRGPI. In both the TCGA and METABRI data sets, we observed that IRGPI was negatively correlated with both PD-1 and PD-L1 expression, and the expression of PD-1 and PD-L1 was positively correlated with CCL5 and CCL25 expression. These results were reproducible, even when we used the semi-quantitative CCL5 and CCL25 IHC scores to calculate IRGPI IHC scores. And we observed the negative correlation between IRGPI IHC scores and PD-L1 expression. More interestingly, IRGPI serum scores, which were calculated with the serum CCL5 and CCL25 concentrations, also showed a close-to-significant negative correlation with tumor PD-L1 IHC scores (*n* = 40, *p* = 0.058). Further validation of these results in a cohort with a large sample size may provide a non-invasive way to test PD-L1 expression in TNBC patients.

Although we repeatedly observed a positive correlation between PD-L1 and CCL5 or CCL25 expression, the molecular mechanisms behind these observations remain elusive. In colorectal cancer, tumor-infiltrated macrophages secreted CCL5, which activates p65/STAT3 pathway and indirectly stabilizes PD-L1 protein rather than increasing the mRNA expression of PD-L1 in cancer cells [77]. And in our study, the TCGA and METABRIC gene expression data are both mRNA expression values. Yet, there are no reports about the regulatory relationship between CCL25 and PD-L1. Nevertheless, IRGPI is a useful and reproducible measurement for PD-L1 expression in TNBC.

## 5. Conclusions

Taken together, we discovered the IRGPI was composed of only two genes. IRGPI is superb in predicting TNBC survival and is a measurement for the infiltration of major players of tumor-immune microenvironmental cells and the status of TILs, as well as PD-1 and PD-L1 expression. IRGPI-low patients may benefit more from the activation of CTLs in ICI therapy.

## Figures and Tables

**Table 1:** Relationship between IRGPI and the clinicopathological characteristics of TNBC patients from our hospital.

Clinical Variables	IRGPI-High (*n* = 40)		IRGPI-Low (*n* = 49)		
	*n*	%	*n*	%	*p* Value
Age, years					0.855
≤50	22	55	26	53	
>10	18	45	23	47	
TNM					0.264
I	11	27.5	20	41	
II–III	29	72.5	29	59	
Grade					0.775
1	0	0	1	2	
2–3	31	77.5	40	82	
Unknown	9	22.5	8	16	
Menopausal status					0.938
Post-	24	60	29	59	
Pre-	16	40	20	41	
Ki-67					0.332
>20%	32	80	34	69	
≤20%	8	20	15	31	

**Table 2:** Univariate and Multivariate Cox analysis of prognostic factors for TNBC patients from our hospital.

Clinical Variables	Univariate Analysis		Multivariate Analysis	
	HR (95% CI)	*p* Value	HR (95% CI)	*p* Value
Age, years				
(>50 vs. ≤50)	0.484 (0.248–0.948)	0.034 *	0.450 (0.229–0.885)	0.021 *
TNM				
II-III vs. <I	1.482 (0.715–3.076)	0.290		
Grade				
1 vs. 2–3	0.748 (0.511–1.097)	0.137		
Menopausal status				
Post- vs. Pre-	0.820 (0.415–1.620)	0.568		
Ki-67				
>20% vs ≤20%	1.101 (0.518–2.342)	0.803		
IRGPI				
Low risk vs. High risk	0.508 (0.263–0.981)	0.044 *	0.470 (0.242–0.912)	0.026 *

* *p* < 0.05.

**Table 3:** Correlations between CCL5/CCL25 and PD-L1 expression.

Pearson Correlation Analysis	CCL5 Expression	CCL25 Expression
PD-L1 expression		
r	0.179	0.203
*p*	0.043	0.021

CCL5, CCL25, and PD-L1 expression was determined by their IHC scores.

**Table 4:** Correlations between IRGPI IHC and serum scores and PD-L1 expression.

Pearson Correlation Analysis	IRGPI IHC Score	IRGPI Serum Score
PD-L1 expression		
r	−0.204	−0.302
*p*	0.020	0.058

IRGPI IHC scores and serum scores were calculated with CCL5 and CCL25 IHC scores or serum CCL5 and CCL25 concentrations. PD-L1 expression was determined by PD-L1 IHC scores.

**Table 5:** Relationship between serum CCL5/CCL25 and PD-L1 expression.

Pearson Correlation Analysis	Serum CCL5	Serum CCL25
PD-L1 expression		
r	0.148	0.286
*p*	0.362	0.073

## Data Availability

The data sets used and/or analyzed during the current study are available from the TCGA database (https://tcga-data.nci.nih.gov/, accessed on 11 January 2021) and the cbioportal website for METABRIC database (https://www.cbioportal.org/, accessed on 12 August 2021). Other data could be available from the corresponding author on reasonable request.

## Supplementary Materials

The following are available online at https://www.mdpi.com/article/10.3390/cancers13215342/s1, Figure S1: Identifying co-expressed immune genes related to CC chemokine genes by WGCNA. (a) Selection of Soft Threshold in WGCNA analysis; (b) gene distribution in WGCNA network analysis; (c) co-expression network of genes in the brown module; (d) GO enrichment of genes in the brown module of co-expression network; (e) KEGG enrichment of genes in the brown module of co-expression network. Figure S2: Validation of the prognostic value of IRGPI. (a) Univariate and multivariate Cox analysis of clinicopathological factors and the IRGPI scores (*p* < 0.05); (b) effect of CCL5 and CCL25 expression on the survival in TNBC cohort of Kaplan–Meier plotter database. Figure S3: Validation of the prognostic value of IRGPI and its associations with immune characteristics in METABRIC database. (a) Kaplan–Meier survival analysis of IRGPI scores using METABRIC data (*p* < 0.05); (b) the difference of immune cell infiltration in different IRGPI groups (pink: IRGPI-high group; and blue: IRGPI-low group); (c) scores of TIDE, MSI, and T-cell exclusion and dysfunction in different IRGPI groups (ns: not significant, * *p* < 0.05; ** *p* < 0.01; *** *p* < 0.001); (d) correlations between CCL5/CCL25/ IRGPI scores and PD-L1/PD1 expression. Figure S4: Pictures of CCL5, CCL25, and PD-L1 immunohistochemistry using the known tissue positive for CCL5, CCL25, and PD-L1. (a) CCL5 IHC in tonsil; (b) CCL25 IHC in thymus; (c) PD-L1 IHC in tonsil. Table S1: Genes in the blue and brown modules. Table S2: DEGs in TNBC and normal samples. Table S3: The 67 immune-related hub DEGs. Table S4: The 41 KEGG pathways of 67 immune-related hub DEGs. Table S5: DEGs in different IRGPI groups. Table S6: CIBERSORT results. Table S7: TIDE results.
