# Supplementary material for: An Immune-Related Gene Prognostic Index for Triple-Negative Breast Cancer Integrates Multiple Aspects of Tumor-Immune Microenvironment
Source: Cancers (Basel). 2021 Oct 25;13(21):5342. doi: 10.3390/cancers13215342 (PMC8582543; doi:10.3390/cancers13215342)
Supplement: Supplementary file 1 [file cancers-13-05342-s001.zip › cancers-1439956-supplementary.pdf]

Article

An Immune-Related Gene Prognostic Index for Triple-Negative Breast Cancer Integrates Multiple Aspects of Tumor-Immune Microenvironment

Xiaowei Wang [1], Wenjia Su [2], Dabei Tang [1], Jing Jing [1], Jing Xiong [3], Yuwei Deng [1], Huili Liu [1], Wenjie Ma [1], Zhaoliang Liu [4,5,*] and Qingyuan Zhang [1,*]

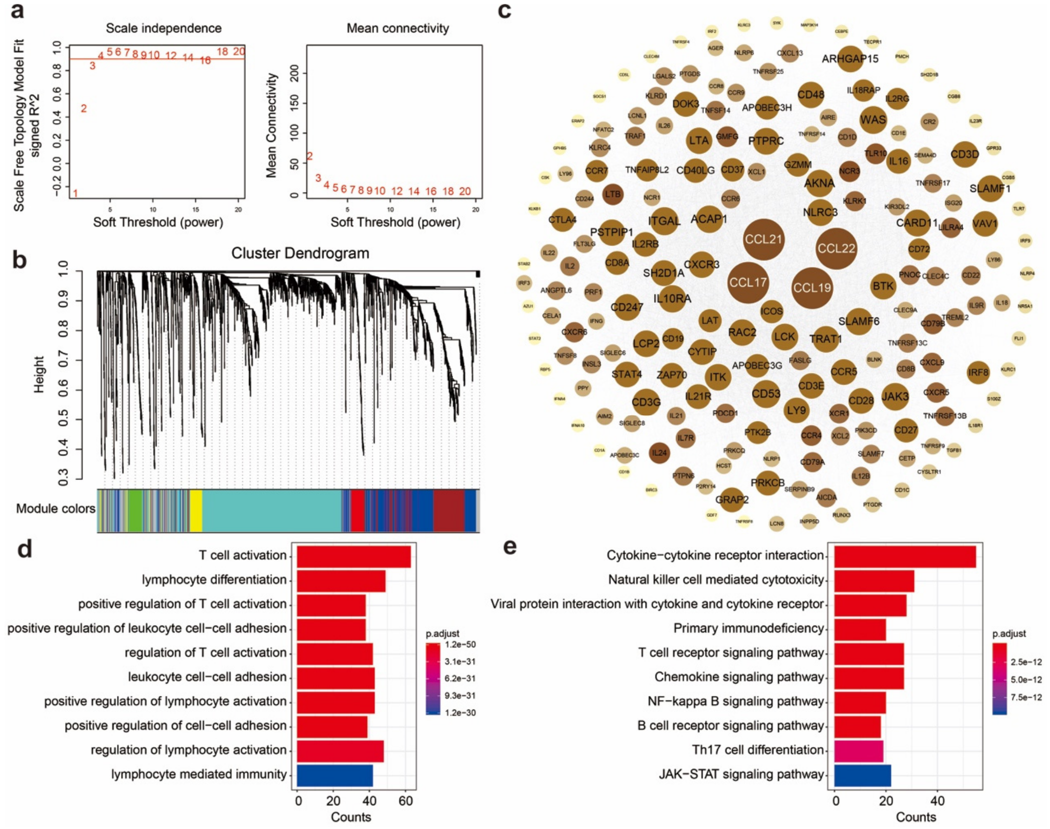

Figure S1. Identify co-expressed immune genes related to CC chemokine genes by WGCNA. (**a**) Selection of Soft Threshold in WGCNA analysis; (**b**) Gene distribution in WGCNA network analysis; (**c**) Co-expression network of genes in the brown module; (**d**) GO enrichment of genes in the brown module of co-expression network; (**e**) KEGG enrichment of genes in the brown module of co-expression network.

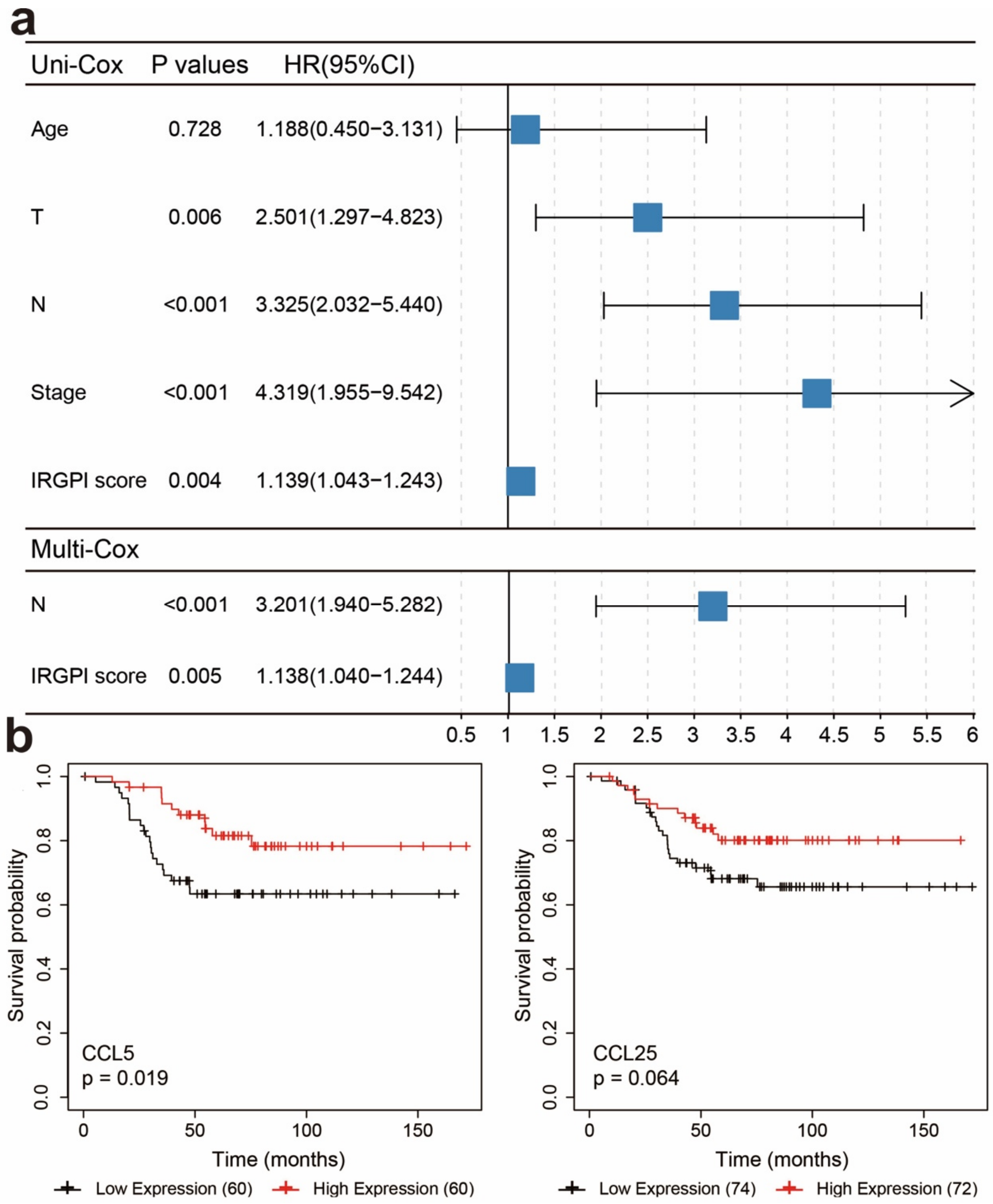

Figure S2. Validation of the prognostic value of IRGPI. (**a**) Univariate and multivariate Cox analysis of clinicopathological factors and the IRGPI scores (p <0.05); (**b**) Effect of CCL5 and CCL25 expression on the survival in TNBC cohort of Kaplan-Meier plotter database.

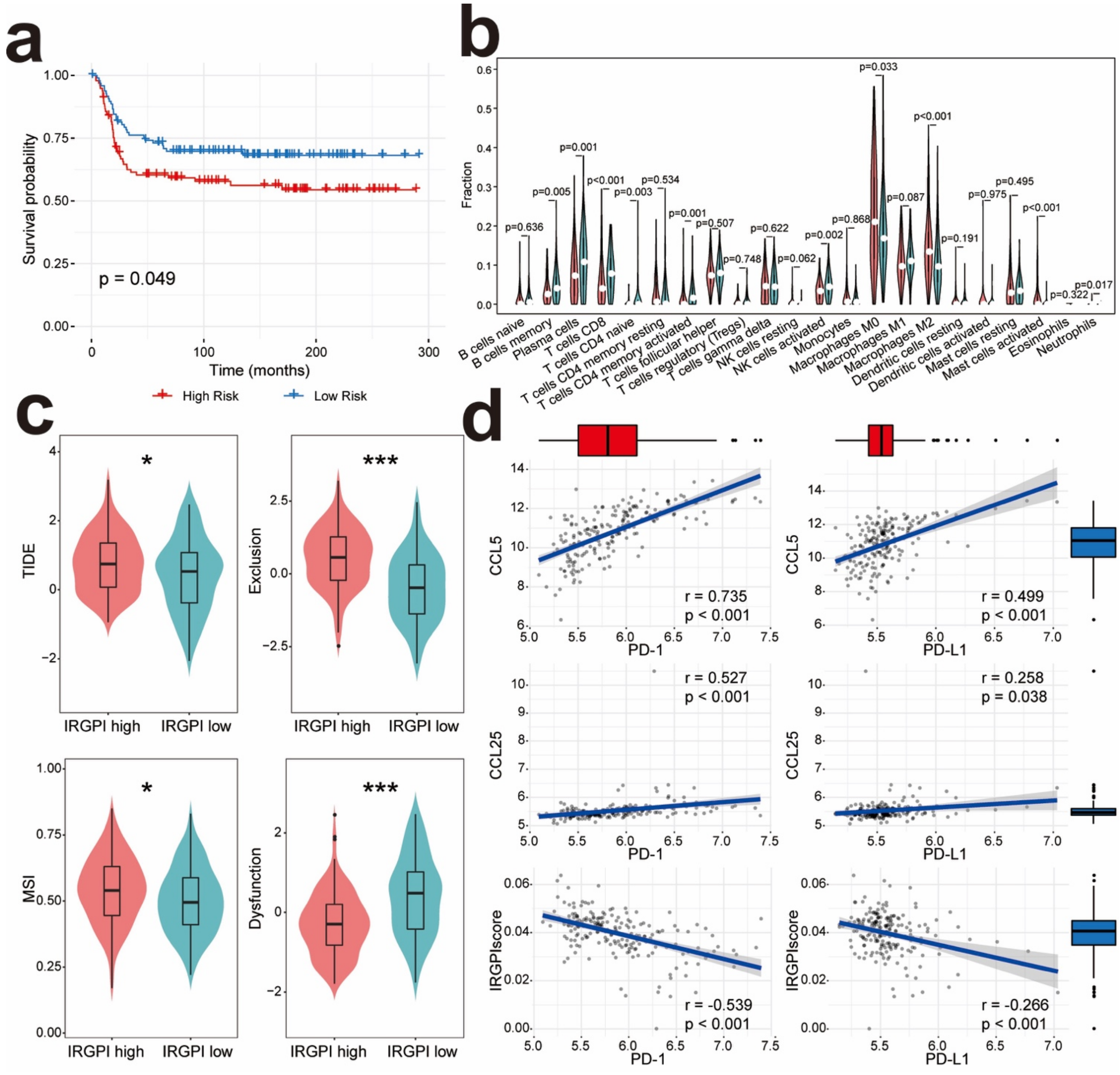

Figure S3. Validation of the prognostic value of IRGPI and its associations with immune characteristics in METABRIC database. (**a**) Kaplan-Meier survival analysis of IRGPI scores using METABRIC data (p <0.05); (**b**) The difference of immune cell infiltration in different IRGPI groups (pink: IRGPI-high group; and blue: IRGPI-low group). (**c**) Scores of TIDE, MSI, and T cell exclusion and dysfunction in different IRGPI groups (ns: not significant, *p< 0.05; **p< 0.01; ***p < 0.001); (**d**) Correlations between CCL5/CCL25/ IRGPI scores and PD-L1/PD1 expression.

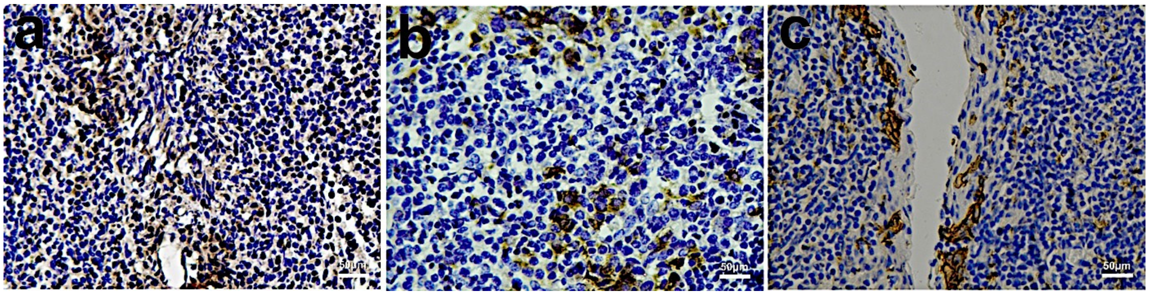

Figure S4. Pictures of CCL5, CCL25 and PD-L1 immunohistochemistry using the known tissue positive for CCL5, CCL25, and PD-L1. (**a**) CCL5 IHC in Tonsil; (**b**) CCL25 IHC in Thymus; (**c**) PD-L1 IHC in Tonsil. Scale bar: 50μm.
